# HER2 (ErbB2) receptors, a potential therapeutic target in squamous cell carcinoma of oesophagus

**DOI:** 10.1038/sj.bjc.6603080

**Published:** 2006-04-18

**Authors:** A N Khan, W Yang, A M Seifalian, M C Winslet

**Affiliations:** 1 Academic Division of Surgical and Interventional Sciences, Royal Free and University College Medical School, University College London, London NW3 2PF, UK

**Sir**,

We read with great interest a recent article by Gibault *et al* (2005) regarding the role of various molecular markers in squamous cell carcinoma of oesophagus (SCCO). We agree with their conclusions that ErbB1 receptors are involved in oesophageal carcinogenesis and prognosis, thus they may be potential targets for immunotherapy of SCCO. However, we have a different view on one of the issues addressed by the group regarding the role of HER2 (ErbB2) in SCCO. In their article, they suggested that their results are consistent with other studies but note a lack of comparative data simply because, to their knowledge, studies concerning ErbB2 expressions are very scarce since a study reported by Shiga *et al* (1993). They therefore concluded that HER 2 (ErbB2) receptors nevertheless appear to be of poor interest as potential therapeutic targets in SCCO.

Traditionally ErbB1 has been considered to be associated with SCCO and majority of the studies have shown it to be overexpressed and associated with poor prognosis in SCCO. However, there is growing body of evidence showing ErbB2 is also abnormally expressed in SCCO and associated with poor prognosis. We reviewed studies reported since 1993 that have analysed ErbB2 expression in SCCO in association with outcome of the disease. Six studies have analysed ErbB2 overexpression in SCCO using immunohistochemistry and ErbB2 has been found to be overexpressed in 9% of cases in one study (Sunpaweravong *et al*, 2005) and 26–64% of cases in the other five studies (Hardwick *et al*, 1997; Friess *et al*, 1999; Wang *et al*, 1999; Akamatsu *et al*, 2003; Mimura *et al*, 2005b). This rate of over expression is greater than the one reported by Gibault *et al* (2.8%), suggesting that ErbB2 is overexpressed to a greater extent in SCCO. Of the six studies described above, two reported a statistically significant association of ErbB2 overexpression with poor prognosis (Mimura *et al*, 2005b; Sunpaweravong *et al*, 2005). ErbB2 overexpression has also been shown to be a marker of chemoradioresistance (Akamatsu *et al*, 2003). Two studies, since 1993 have detected ErbB2 mRNA expression using PCR and reported overexpression in 25 and 28% of cases (Tanaka *et al*, 1997; Miyazono *et al*, 2004). In these two studies ErbB2 overexpression has been associated with extramucosal tumour invasion and poor response to chemoradiation.

Although methodologies used in these studies to detect ErbB2 expression are different but all of them clearly suggest that ErbB2 receptors are overexpressed in SCCO to a greater extent as reported by Gibault *et al*. ErbB2 overexpression has also been associated with invasive disease, poor response to treatment and outcome. Early preclinical studies using Herceptin (anti HER 2 monoclonal antibody) in SCCO cell lines have shown that it does have inhibitory effect on growth of cells, either alone or in combination with conventional treatments (Mimura *et al*, 2005a; Sato *et al*, 2005). On the basis of current evidence, which suggests abnormalities of ErbB2 expression, its association with poor prognosis and evidence that targeting it could be of therapeutic benefit in SCCO, we cannot exclude the possibility of significant role of HER 2 receptors in oesophageal squamous cell carcinogenesis, disease progression and its potential value as therapeutic target, along with ErbB1.

ErbB2-targeted therapies are still in an early stages of development in reference to SCCO and at this stage we look forward to results evaluating its effects in other cancers, where these therapies are in a relatively advanced stages of development. We hope that further research in this field will help determine the value of ErbB 1 and ErbB2 targeted therapies in SCCO.
